# Implementation of Virtual Reality in Health Professions Education: Scoping Review

**DOI:** 10.2196/41589

**Published:** 2023-01-24

**Authors:** Silje Stangeland Lie, Nikolina Helle, Nina Vahl Sletteland, Miriam Dubland Vikman, Tore Bonsaksen

**Affiliations:** 1 Department of Health Faculty of Health Studies VID Specialized University Stavanger Norway; 2 Department of Nursing Faculty of Health Studies VID Specialized University Bergen Norway; 3 Department of Health and Nursing Science Faculty of Social and Health Sciences Inland Norway University of Applied Sciences Elverum Norway

**Keywords:** implementation, virtual reality, higher education, medical education, health professions education, continuing education, scoping review, health professional, technology

## Abstract

**Background:**

Virtual reality has been gaining ground in health professions education and may offer students a platform to experience and master situations without endangering patients or themselves. When implemented effectively, virtual reality technologies may enable highly engaging learning activities and interactive simulations. However, implementation processes present challenges, and the key to successful implementation is identifying barriers and facilitators as well as finding strategies to address them.

**Objective:**

This scoping review aimed to identify the literature on virtual reality implementation in health professions education, identify barriers to and facilitators of implementation, and highlight gaps in the literature in this area.

**Methods:**

The scoping review was conducted based on the Joanna Briggs Institute Evidence Synthesis methodologies. Electronic searches were conducted in the Academic Search Elite, Education Source, and CINAHL databases on January 5, 2022, in Google Scholar on February 2 and November 18, 2022, and in PubMed database on November 18, 2022. We conducted hand searches of key items, reference tracking, and citation tracking and searches on government webpages on February 2, 2022. At least 2 reviewers screened the identified literature. Eligible studies were considered based on predefined inclusion criteria. The results of the identified items were analyzed and synthesized using qualitative content analysis.

**Results:**

We included 7 papers and identified 7 categories related to facilitators of and barriers to implementation—collaborative participation, availability, expenses, guidelines, technology, careful design and evaluation, and training—and developed a model that links the categories to the 4 constructs from Carl May’s general theory of implementation. All the included reports provided recommendations for implementation, including recommendations for careful design and evaluation, training of faculty and students, and faculty presence during use.

**Conclusions:**

Virtual reality implementation in health professions education appears to be a new and underexplored research field. This scoping review has several limitations, including definitions and search words, language, and that we did not assess the included papers’ quality. Important implications from our findings are that ensuring faculty’s and students’ competence in using virtual reality technology is necessary for the implementation processes. Collaborative participation by including end users in the development process is another factor that may ensure successful implementation in higher education contexts. To ensure stakeholders’ motivation and potential to use virtual reality, faculty and students could be invited to participate in the development process to ensure that the educational content is valued. Moreover, technological challenges and usability issues should be resolved before implementation to ensure that pedagogical content is the focus. This accentuates the importance of piloting, sufficient time resources, basic testing, and sharing of experiences before implementation.

**International Registered Report Identifier (IRRID):**

RR2-10.2196/37222

## Introduction

### Research on Virtual Reality

The implementation of technology is slow in higher education because of barriers to technology use, and the sharing of innovative and successful practices appears to be lacking [1]. This led to our interest in exploring how virtual reality is implemented in health professions and continuing education and which success factors exist.

Virtual reality is a broad concept. In the research literature, the term encompasses several categories: screen-based virtual reality, virtual worlds, and immersive virtual reality environments [2]. In this study, we defined virtual reality as a digital representation of a 3D environment. We focused on immersive virtual reality, wherein head-mounted displays are used to block out the real world, which coincides with the general understanding of what constitutes virtual reality [3-5]. Such virtual reality applications in higher education hold great promise for supporting students’ learning and providing positive experiences in education programs [6]. They may also provide health care students with a platform to experience and master situations without endangering patients or themselves [7,8].

Until recently, virtual reality has mostly been used in technical higher education (eg, engineering, computer science, and astronomy) [9]. However, the use of virtual reality in health professions education is gaining ground and is starting to play an important role in competence development. Immersive technologies can provide learning gains similar to those provided by traditional educational modalities [10]. They can increase attention and enhance skills and confidence and seem to influence users’ emotional responses to learning situations, which in turn increases learning motivation [11]. Furthermore, other outcomes, such as student satisfaction, self-efficacy, and engagement, may increase when using such technology, suggesting that it is a viable tool in health professions education [10]. A systematic review from 2021 examined the use of virtual reality to train nursing students and found it to be a feasible teaching strategy to improve knowledge acquisition when used to supplement, but not replace, conventional teaching methods [8]. Another systematic review concluded that virtual reality that aims to train health care professionals in soft skills (eg, teamwork and communication) is gaining ground as a promising prospect for health care professionals’ continuing education [2]. When implemented effectively, virtual reality technologies enable highly engaging learning activities and interactive simulations [12].

Although recent research supports the use of virtual reality training in the context of health professions education, it also presents new challenges [8]. Several researchers have reported that students found virtual reality implementation to be insufficiently realistic, alleging that this was a result of the limited time and available resources [4]. For faculty and students to use innovative technology in training, new ways of working are required for both parties. Therefore, implementing virtual reality requires changes to the organization or system within which the implementation is planned. To ensure successful implementation, it is necessary to identify barriers and facilitators as well as strategies to address them [13]. More and higher-quality research studies are required to explore the acceptability and effective implementation of this technology [11]. Thus far, qualitative studies have suggested easier uptake and more positive experiences among students with a high affinity toward technology [14], indicating that successful implementation relies on organizational as well as individual readiness.

Literature searches conducted for our study protocol [1] identified reviews concerning virtual reality in higher education, some of which reported on virtual reality in health professions education [2,8,15]. Virtual reality simulation training for disaster preparedness in hospitals has been covered by an integrated review [16]. The search also identified a scoping review protocol on virtual reality education for dementia care [17] and an integrative review on the applications of and challenges of implementing artificial intelligence in medical education [18]. However, no current or in-progress scoping review or systematic review reporting on virtual reality implementation in health professions education was identified [1]. To address this literature gap, this scoping review set out to identify literature on virtual reality implementation in health professions education to identify barriers to and facilitators of implementation as well as to highlight research gaps in this area.

### Research Question

What recommendations for the implementation of virtual reality in health professions education are provided in the available literature?

### Theoretical Background

In this paper, we define implementation as “the act of putting a plan into action or starting to use something” [19]. The implementation and embedding of innovative technology in higher education occurs in complex organizational environments, but other demands from busy work schedules may undermine this novel task. People need motivation to make things happen, such as using innovative technology such as virtual reality and changing their educational practices. The purpose of Carl May’s general theory of implementation “is to help facilitate both prospective understanding of implementation processes and evaluation of their outcomes” [20]. This theory is intended to be a starting point for understanding and evaluating the implementation of complex interventions in health care practice. We found it conducive to use this theory in the higher education context, as this is also a complex organizational environment with many actors and systems involved. According to May’s theory, 4 constructs may be crucial for effective virtual reality implementation—*capacity, potential, capability, and contribution—*which concern both planning the implementation process and evaluating its progress and outcomes [20].

Virtual reality implementation in health professionals’ education depends on faculty’s and students’ *capacity* to change their interactions as well as their assumed capability to use virtual reality. Social norms, roles, and material and cognitive resources are required to operationalize the intervention. Norms and roles are affected when incorporating innovative technology, such as virtual reality, into a social system (ie, the educational program in question). Moreover, informational and material resources shape practice and participants’ accountabilities, influencing their capacity to use virtual reality. *Potential* concerns agency and motivation, which are antecedents of the dynamic and emergent conditions that follow virtual reality implementation. Individuals’ intention and personal interest in virtual reality are important, but even more important is that the members of the organization collectively value the changes that the implementation process will elicit. If they value it enough, they will be committed to it. Individuals’ intentions and shared commitment create readiness for virtual reality implementation. *Capability* concerns the workability of the technology at hand and the integration of the system into the given context. In this setting, capability concerns the ensembles of behaviors and practices around virtual reality objects and the procedures required to use virtual reality in education. Finally, *contribution* concern*s* how virtual reality implementation is a collective, coordinated, and collaborative social action. Joint action is necessary for the successful implementation of virtual reality in educational settings. When the involved actors contribute to implementation, they perform directed actions and perform the practices required to implement and embed virtual reality in their contexts. When actors agree on the technology and value it, they gain cognitive authority and their actions become meaningful, which are crucial to the implementation process [20].

## Methods

### Context

A challenge when implementing technologies such as virtual reality in higher education is to diminish the barriers’ effects and enhance the facilitators’ effects. Therefore, during the development phase of an educational project [21], we undertook this scoping review to systematically map the virtual reality implementation literature related to health professions education and to identify key concepts and sources concerning implementation, along with any literature gaps [22]. Considering that research on virtual reality implementation in health professions education is novel and groundbreaking, we present recommendations for the implementation of virtual reality in this setting. The scoping review has been reported based on the PRISMA (Preferred Reporting Items for Systematic Reviews and Meta-Analyses)–Extension for Scoping Reviews checklist (Multimedia Appendix 1) [23].

### Literature Search

Keyword search refinement was conducted from November to December 2021 and is reported in the scoping review protocol [1]. A systematic literature search was performed on January 5, 2022, in the following databases: Academic Search Elite, Education Source, and CINAHL. Three keywords were used—“virtual reality,” “higher education (health),” and “implementation”—as well as several synonyms and medical subject heading terms. The keywords were combined with “AND.” We performed an additional search in PubMed on November 18, 2022, using the same keywords and medical subject heading terms. Refer to Multimedia Appendix 2 for the search strategy used.

The inclusion criteria for the search comprised articles published within the past 5 years (2017-2022); articles concerning higher education or health professions education, including medicine and continuing education; articles examining a particular age group (>18 years); articles concerning virtual reality or virtual reality simulation aspects; and articles written in English.

In Google Scholar, the following search combination was used on February 2, 2022: “implement* virtual reality health professional higher education,” which was limited to articles published after 2017. This yielded 17,000 hits. The first author screened the first 50 articles, resulting in the identification of 3 (6%) articles that qualified for further screening [9,24,25]. Furthermore, we conducted hand searches of key items, reference tracking, and citation tracking, eliciting 1 article that qualified for further screening [8]. The first author performed an updated search in Google Scholar on November 18, 2022, which was limited to articles published in 2022. This yielded 16,900 results, of which the first 50 were screened. No new articles relevant to this review were identified through this search.

Through the literature search performed in January and February, 404 articles were included after duplicates were removed. The authors screened these articles (titles and abstracts) based on the inclusion criteria. Blind screening was conducted using the Rayyan (Rayyan Systems Inc) web tool [26], and at least 2 authors screened each article. The first author screened all 404 articles, and the coauthors divided the articles among themselves to ensure double screening of all articles. Before the screening process, we piloted the screening of 1.3% (5/404) of randomly chosen articles to ensure a similar understanding of the inclusion and exclusion criteria. This further aided in the screening process. Moreover, after screening the titles and abstracts, we discussed articles regarding which the decisions of the 2 authors who screened those were conflicting (17/404, 4.2%). After reaching a consensus based on the inclusion and exclusion criteria, 6.5% (26/404) of articles were included for full-text reading. An additional PubMed search conducted in November 2022 yielded 94 articles for screening. On the basis of the inclusion and exclusion criteria, 7% (6/94) of these were included for full-text reading, in addition to the previous 26 articles.

The first author conducted hand searches for white literature on Norwegian government web pages on February 18, 2022. The decision to search only Norwegian documents was made because of this project’s placement in a Norwegian higher education institution. The Norwegian keywords used in the search were “Implement*,” AND/OR “virtual reality” (as the English term is commonly used in Norwegian), AND “teknologi”; the search included papers published in the past 5 years. Three white papers were identified through these hand searches and included for full-text reading, along with 32 articles identified through literature searches of the databases. We considered eligible studies based on the criteria presented in Textbox 1.

**Inclusion and exclusion criteria.**

**Inclusion criteria**

- Participants: students, faculty, and health care professionals (adults)
- Concept: implementation of virtual reality
- Context: higher and continuing health professions education

**Exclusion criteria**

- Flatscreen simulation or 2D videos
- Use for patients, clinicians, and children

### Data Analysis

Following guidance for completing scoping reviews [27,28], all the authors extracted the following data from the included papers in a matrix before synthesis: author and country of origin, year of publication, aims and purpose, study population, methodology and sample description, concept, outcomes, and key findings related to the research objectives. The data extraction tool has been reported in our review protocol [1]. Data synthesis was conducted using qualitative content analysis [29]. First, the data were sorted according to the 3 factors in the data extraction form (facilitators, barriers, and recommendations). Second, the texts were grouped according to similarities and differences, and tentative categories were created. The categories were revised several times, and the content was shifted back and forth between categories until the authors reached a consensus on 7 categories that described the data’s manifest content. Thus, the categories describe recommendations for virtual reality implementation in accordance with the research question.

## Results

### Overview

**Figure 1:**
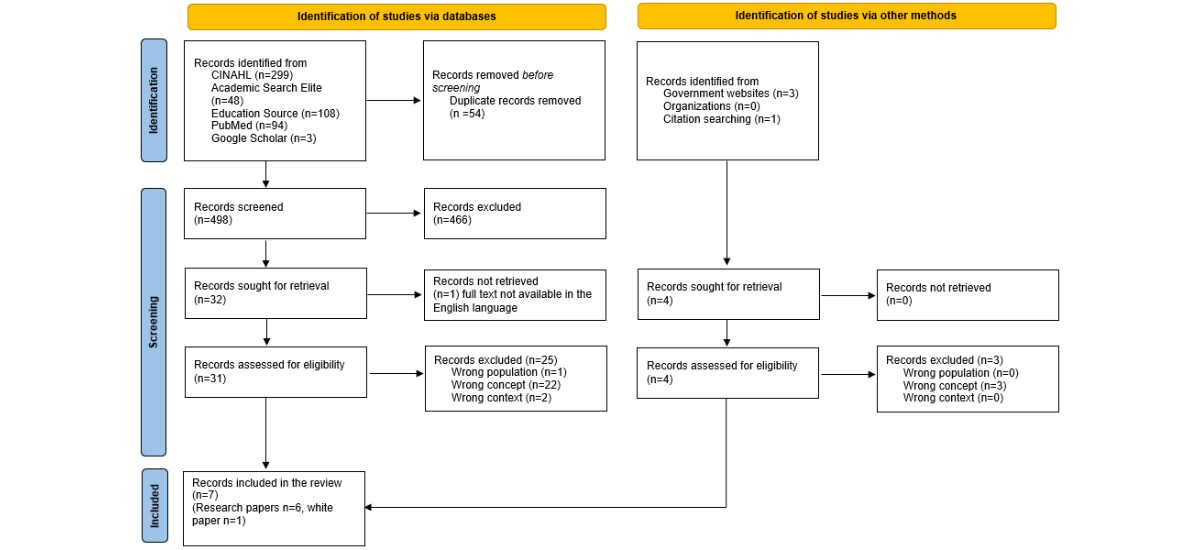
PRISMA (Preferred Reporting Items for Systematic Reviews and Meta-Analyses) flow diagram of the inclusion process.

Figure 1 is a PRISMA flow diagram that lays out the search and inclusion process [28]. It contains the results from the initial literature search conducted in January and February 2022 as well as those from the additional search conducted in November 2022. We included 7 (1.4% of the total 498 records screened) papers [30-36], and the key information from these papers is presented in Table 1.

By conducting a content analysis of the data extracted from the included articles, we identified seven categories that describe the recommendations for virtual reality implementation provided in the included literature: (1) collaborative participation, (2) availability, (3) expenses, (4) guidelines, (5) technology, (6) careful design and evaluation, and (7) training. These categories relate to both the facilitators of and barriers to implementation and are described in detail in the subsequent sections to coordinate the findings and recommendations from the included articles.

**Table 1:** Summary of the included records.

Reference and country of origin	Method for data collection and analysis	Participants, setting, and response rate if stated	Key findings		
			Facilitators of VR^a^ implementation	Barriers to VR implementation	Recommendations for VR implementation
D’Errico [32], 2021, the United States and Canada	N/A^b^	One nursing simulation educator and a group of VR simulation software developers met within the VR environment	VR facilitates connections and collaborative engagement Joint VR experiences facilitate problem resolution and identification of what works	Equipment must be available and meet the required standards Appropriate technological infrastructure is required for new equipment to work	Using the VR environment during the implementation process is a good way to promote team collaboration, design and test realistic scenarios, and identify and resolve problems within VR
Rim and Shin [34], 2020, Republic of Korea	Two-phase methodological study design: (1) developing a preliminary template and (2) evaluating its usability through focus group interviews Content analysis	n=16 students Two focus group interviews with 8 students each	Repeated practice improves nursing ability through the following: Improved confidence Exposure to patient situations enables participants to adapt to new situations Using an active avatar provides a sense of reality	Technological difficulties Insufficient time	Secure competent human resources as well as the capabilities that they require Develop and apply templates or frameworks, including the following: Training time Determining the overall objectives through expected outcomes Prelearning, prebriefing, and debriefing sessions Evaluation Incorporate technology into VR, including artificial intelligence for programmed patients, to increase learners’ sense of presence, affordance, and immersion
Saab et al [35], 2021, Ireland	Qualitative descriptive study using thematic analysis	n=26 students Undergraduate nursing students participated in face-to-face, semistructured individual interviews and focus groups	An available human facilitator to supervise and guide students before, during, and after VR use VR used in small student groups VR equipment was available for students to borrow	Time and cost: VR takes more time with larger class sizes Cost of equipment Not suitable for several people simultaneously owing to expense Human resources required to convert the current material to VR Physical limitations to use: Sight problems, vertigo, dizziness, motion sickness, and risk of injury	Background knowledge before lecture or practice in using VR is needed Secure a sufficient number of VR headsets Create an appreciation of difficulties (eg, hearing or sight impairments): Offer VR educational experience on a standard desktop for individuals who experience motion sickness VR is suitable for supplementing conventional teaching and learning methods but not as a stand-alone approach Address issues such as technology costs, space, and training in VR use
Baniasadi et al [30], 2020, Iran	Literature review	Medical students and treatment context	Usable and user-friendly VR approaches Developing and updating related laws, guidelines, and standards Using appropriate models in design and implementation	Cost of equipment, design, and implementation Lack of knowledge about, competence in, and trust in technology Difficulties in providing content Organizational culture Lack of management support	Manuals and training for end users User participation in the design process Due to the lack of face-to-face communication between students and real patients when using VR for training, evaluations should be made in real settings to ensure efficacy
Barteit et al [31], 2021, Germany, the United States, South Africa, and Zambia	Systematic review, PRISMA^c^	n=27 health professionals in medical education Evaluation methods comprising practical skill tests Most included studies evaluated the head-mounted displays’ efficacy	Head-mounted displays offer the possibility of scalability and repeated practice, such as in the following: Practical procedures Anatomy Developing communication-skills	The context for effective implementation: The individual learner The learning environment The learning implementation’s context The technological environment The pedagogics involved	Implementation of Miller’s Pyramid of Professional Competence undergirds XR^d^-based HMD’s^e^ potential A framework or guidelines for XR-based HMD interventions are needed to guide implementations and evaluations
Kunnskapsdepartementet [33], 2021, Norway	A government document and background paper	Case drawn from an exemplary Norwegian University	The VR laboratory was open for students 24/7 VR laboratories enable students to practice examining patients and interacting with others in clinical situations	Educational institutions cannot deviate from the requirements of the EU’s^f^ Vocational Qualifications Directive (because of the EEA^g^ agreement); these requirements hinder replacing clinical practice with simulation in nursing education	The Norwegian government encourages more VR simulation in education regulated by the directive than what is possible today VR simulation might replace clinical practice
Hood et al [36], 2021, Australia	Case study reporting on initiation, concept design, pilot implementation, and feasibility assessment of a VR training platform	Pilot implementation at 7 hospitals User survey: n=61 in the pretraining survey and n=58 in the posttraining survey Logging use sessions	TACTICS VR was delivered in the context of a broader education implementation trial The VR training program was specifically designed to promote user interactions and active learning (eg, interactive elements and gamification) to promote user engagement and maximize the benefits of using VR technology VR deployment was supported by on-site trial coordinators at each hospital	The pilot implementation identified problems or issues with Wi-Fi connectivity across multiple hospitals’ IT systems	The Wi-Fi connectivity issue was overcome by supplying mobile Wi-Fi routers to maintain connectivity Site coordinators suggested that additional implementation approaches could increase training reach (eg, integration into the existing clinical training programs)

^a^VR: virtual reality.

^b^N/A: not applicable.

^c^PRISMA: Preferred Reporting Items for Systematic Reviews and Meta-Analyses.

^d^XR: extended reality.

^e^HMD: head-mounted display.

^f^EU: European Union.

^g^EEA: European Economic Area.

### Collaborative Participation

Overall, 3 concepts of collaboration were described in the included literature. *Collaboration in the design* of the virtual reality system, including user participation (students and faculty) in the design process, was essential to create usable and user-friendly applications, helped identify limitations, and played a critical role in successful virtual reality use [30,32]. *Collaboration by developers inside the virtual reality environment* for system design purposes was described by D’Errico [32]. This helped create realism and fidelity as well as identify errors. By being mutually immersed in the virtual world, the design team experienced the scenario together and efficiently identified solutions to problems [32]. The third and last concept of collaboration described was *collaboration inside virtual reality environments* by students (end users). Being able to move freely in the system using an active avatar provided a sense of reality and improved the sense of participation [34]. Such interactive elements promote user engagement and maximize the benefits of using virtual reality technology [36]. Students could practice examining patients, analyzing scenarios, and interacting with others in clinical situations in a virtual reality environment [33]. Such interactivity was described as facilitating implementation.

### Availability

Availability concerns both the availability of virtual reality headsets and that of faculty and support staff during use. Successful virtual reality implementation depended on the suitable scheduling of the education programs [30]. Providing a system that allows students to borrow virtual reality headsets for 10 minutes facilitates the use of virtual reality [35]. Allowing repeated practice [34] and making virtual reality laboratories available to students 24/7 [33] were mentioned as facilitators.

Faculty availability during virtual reality use was also mentioned as being critical. On-site coordinators or facilitators for providing assistance in using the virtual reality system (address questions, brief students, and provide continuous feedback) were described as crucial to successful virtual reality implementation, both in preparation and actual use [34,36]. Moreover, using virtual reality in small tutorial groups rather than during lectures was advised [35].

### Expenses

Virtual reality design and implementation are expensive because it takes time and resources to convert the current material into virtual reality [35]. The supply and costs of equipment are barriers to virtual reality implementation in health professions education [30,32,35]. In addition, virtual reality laboratories require space, which is also an expense for institutions. Training faculty and students to use virtual reality is time-consuming [30,35]. The time element is also crucial to expenses because virtual reality, owing to equipment costs, might not be feasible in large classes, at least not simultaneously [34,35]. Supplying enough equipment was mentioned as a barrier in several of the included articles. One recommendation was to secure sufficient virtual reality headsets so that students would not fall behind [35]. Moreover, Saab et al [35] asserted that virtual reality should supplement conventional teaching, which also affects expenses considerably.

### Guidelines

The reported barriers to successful virtual reality implementation included a lack of suitable standards, insufficient infrastructure, difficulties providing content, organizational culture, and a lack of management support [30]. The need for frameworks or guidelines to help implement virtual reality in health education was mentioned [30,31]. Therefore, developing and updating related laws, policies, guidelines, and standards, as well as using appropriate models in the design and implementation of virtual reality applications, could be beneficial for virtual reality implementation in health education [30,33]. In several European countries, the European Union’s Vocational Qualifications Directive regulates nursing education programs. The directive regulates the duration of students’ clinical placement, hindering the replacement of clinical practice with virtual reality simulation. This may create a barrier to the implementation of virtual reality laboratories in educational institutions. The Norwegian Ministry of Education has described the need for changes in regulations to enable the inclusion of simulation as a larger part of health education. Technical and pedagogical developments make it possible to implement teaching in new ways, with more student-active forms of learning and increased learning as the expected results [33].

### Technology

Technological problems and usability difficulties were mentioned as significant barriers to successful virtual reality implementation [30,35,36]. People’s IT skills (or lack thereof) and unfamiliarity with virtual reality hinder its use. Having a system for identifying and addressing technical limitations plays a key role in implementation processes.

The size, weight, and general clunkiness of the virtual reality headsets hinder some people in their use of the headsets. Others may experience sight problems, vertigo, dizziness, or motion sickness, which can hinder the use of virtual reality [35]. Some virtual reality systems, such as 360º videos, have little or no possibility of interaction or interactivity, which is also viewed as a barrier [32]. Incorporating more advanced technology into virtual reality, such as artificial intelligence and active avatars, to increase learners’ sense of immersion would benefit the overall experience [34].

### Careful Design and Evaluation

The careful design of virtual reality for health education is central [30,32,34,36]. To plan virtual reality training, instructors need to determine the overall objectives based on the expected outcomes. The pedagogics involved in the virtual reality learning experience were mentioned as being important for implementation and comprised the individual learner, learning environment, context, and technology. Rim and Shin [34] recommended a template containing educational elements, virtual elements, and scenario outlines. The educational elements that are important to the planning of virtual reality training are learning objectives, course flow, and feedback strategies. The virtual elements and how they work are also central to the efficient designing of virtual reality. Moreover, careful planning of scenario outlines is crucial, and this includes the scenario, intended learning objectives, evaluation, mechanical support, and debriefing components. Evaluations should be conducted when using virtual reality in educational settings to ensure the program’s efficacy and desired outcomes [34].

### Training

The training of end users was mentioned in several articles as one of the success factors for virtual reality implementation [30,34,35]. Practically using virtual reality, rather than being instructed theoretically, during training is valuable. Moreover, preparing students before use, assisting during use, and debriefing after use are viewed as crucial for successful implementation [34]. According to Barteit et al [31], virtual reality implementation benefits from using the Miller’s Pyramid of Professional Competence—“See one, do one, teach one, and simulate one”—such that students are invited to facilitate simulation, after having participated themselves. Moreover, virtual reality applications in health education require a comprehensive manual that specifies how, where, and for whom this technology is appropriate [30], which is also relevant for providing training in and preparing for virtual reality use.

## Discussion

### Principal Findings

**Figure 2:**
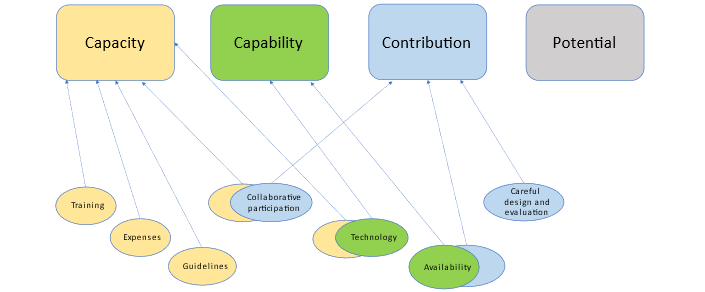
Categories of implementation recommendations mapped onto May’s general implementation constructs.

The purpose of this scoping review was to identify literature reporting on virtual reality implementation in health education and to explore which recommendations for implementation are provided in the available literature. On the basis of a systematic and thorough search and screening process and the inclusion and exclusion criteria presented, 7 papers were included—6 (86%) research articles and 1 (14%) government report. The fact that the number of papers deemed appropriate for inclusion is low indicates that research focusing specifically on virtual reality implementation is scarce. The articles that reported on facilitators focused primarily on human agents preparing for and performing within the virtual reality environment as well as the system’s perceived convenience. Several barriers to virtual reality implementation were mentioned, particularly those concerning expenses, guidelines, and technology. All the included reports provided recommendations for implementation, particularly in the *Conclusion* section. These involve recommendations for careful design and evaluation, the training of faculty and students, and the presence of faculty during virtual reality use (as is also described under facilitators). Our model (Figure 2) links our categories to May’s 4 constructs [20]. We have discussed our findings in the following section, considering both theory and earlier research.

### Comparison With Prior Work

#### Capacity

Our findings indicate that *training* for competency development is vital for enabling successful implementation and ensuring competent use among both students and faculty. Training as a prerequisite for successful virtual reality implementation relates to May’s construct capacity [20], as it is necessary for both parties to have the capacity to use virtual reality. Our findings indicate that it could be useful for students to first observe, then conduct, and thereafter teach fellow students how to use virtual reality simulations to obtain the necessary skills and confidence to use virtual reality gradually [31].

The faculty’s lack of technological competence was mentioned as a barrier to successful virtual reality implementation in education [37]. According to May [13], norms and roles are affected when innovative technology is incorporated into an educational context. We may speculate regarding whether students have a greater capacity to use innovative technology than the faculty because most students within “Generation Z” are digital natives. This could affect social roles and norms and even change the power dynamics in the classroom setting. For example, when using virtual reality in simulations, the faculty may need to take on less of an “expert role” and function more as facilitators [38].

However, we should be careful when assuming that all students are equally confident in using innovative technology. Training students and faculty is important because if students do not master virtual reality, they may not enjoy the possible pedagogical benefits that come from using it [39]. Implementing virtual reality in itself does not necessarily promote good teaching and learning for students [40]. Technology needs to be anchored firmly in the pedagogical approach; therefore, the knowledge of students’ and teachers’ training needs and experiences is important when implementing virtual reality. By securing well-planned training, cognitive resources can be ensured [20].

Related to this, and as our findings indicate, facilitating *collaborative participation* and providing *guidelines* are crucial for implementation. Guidelines that include informational and material resources provided to users are important and influence users’ capacity to use virtual reality. This corresponds to previous research suggesting that when using virtual reality in nursing education, clear guidelines and objectives for the applications are crucial to ensure successful use [41]. Moreover, virtual reality applications designed with consumer usability in mind are easier to use when training students and implementing virtual reality in higher education settings. Therefore, it is vital to ensure collaborative participation by including end users in the development process [42-44]. This is relevant to all institutions planning to implement virtual reality in their educational programs.

Training, collaborative participation, and the development of guidelines for proficient use can be time-consuming and expensive. Our findings indicate that expenses are a crucial aspect of virtual reality implementation in higher education. Expenses can also be related to May’s *capacity* construct. The supply and cost of equipment and the time and space required for virtual reality implementation are important aspects that need to be considered. When a program contains 300 students, using virtual reality as an educational method for all students is time-consuming, even if the institution has secured as many as 50 virtual reality headsets. Furthermore, storing several virtual reality headsets (eg, in a virtual reality laboratory) demands space in institutions, which also incurs high costs. Therefore, it could be of value to conduct cost-benefit analyses when implementing virtual reality in higher education [44], as Saab et al [35] argued that virtual reality should supplement, but not replace, the conventional teaching and learning methods.

#### Capability

Our findings indicate that availability is crucial and that it is necessary to provide sufficient time for both students and faculty to adjust when implementing and using virtual reality in health professions education. This builds on previous evidence that emphasizes the importance of a generous time window for successful virtual reality use in undergraduate programs [12]. Moreover, our findings suggest that ensuring that virtual reality technology is available to students is essential for its implementation. Woon et al [8] recommended that “virtual reality training should consider self-guided, multiple short sessions in delivering procedural content using low-to-moderate immersion.” However, as mentioned in our findings, the presence of competent and experienced facilitators may be important for students’ potential for learning through virtual reality [34]. Facilitators’ presence is another factor in the *availability* category, which enables facilitators to brief students, answer questions, and provide continuous feedback. This contrasts with the recommendation for self-guided virtual reality use, as mentioned above, and it is important to explore this further in future research.

According to the presented theoretical framework, capability also concerns practices related to implementation [20]. Extant research has found that students prefer using new technologies in education if they make them experience emotions, such as motivation and enthusiasm, as well as provide experimental opportunities [45]. Faculty should strive to ensure that facilitators have the interpersonal, technical, and professional skills to create engaging virtual learning arenas for students [32,35], which may be a challenge. To make virtual reality useful in a higher education context, facilitators need sufficient time and clarification regarding their guiding educational and technical roles. Thorough behavioral and practical training of facilitators may reduce barriers to implementation and facilitate the creation of constructive learning arenas [20]. This can be used to prevent a so-called “implementation gap,” in which a lack of organizational readiness for change can lead to the unsuccessful implementation of new technologies [46].

Technological difficulties (eg, unfamiliarity and usability difficulties for facilitators) and practical barriers that hinder virtual reality users are major implementation barriers. Technological challenges should be resolved before implementation to ensure that pedagogical content is the focus, and not the technical barriers. This accentuates the importance of allotting sufficient time and resources to conduct basic testing and share experiences before implementation begins. It is important that the various parties involved in the process, both technical and educational, conduct constructive dialogues during the process [39]. Our findings indicate that a lack of knowledge about and experience with technology is an obstacle to virtual reality use. This builds on earlier research concerning the implementation of health technology, which concluded that even though users were motivated to learn how to use the new technology, a lack of information, sustainable infrastructure, and available resources hindered its implementation [46]. Our findings demonstrate the importance of having a system in place to identify and address the technical limitations when implementing virtual reality. Therefore, it is vital to develop a clear framework and action plan to address the different foci of the various stakeholders involved in the implementation process as well as to clearly define their roles and responsibilities.

Another barrier to the implementation and successful use of virtual reality is that some students experience sight problems, vertigo, dizziness, and motion sickness (also called virtual reality sickness) [35]. This is also related to the capability concept. Earlier research has described several ways to prevent virtual reality sickness, including moving the body and adding multisensory information (eg, music or aromas) [47]. These suggestions may be of value when planning virtual reality implementation in higher education contexts.

#### Contribution

Our findings concerning collaborative participation, careful design and evaluation, and availability connect with May’s construct contribution [20]. Implementing virtual reality is a collective, coordinated, and collaborative social and joint action in the context of higher education. The implementation of innovative technology depends not only on what can be done but also on the current stakeholders’ attitudes toward and interest in new technological solutions. When the involved actors contribute to the implementation of virtual reality, they perform directed actions, continuously build and act on their functions, and perform the necessary practices to implement and embed virtual reality in their practice. For of the implementation of virtual reality, it is crucial that the actors agree with and value it. This gives participants cognitive authority and adds meaning to their actions [20].

Our findings concerning collaborative participation and availability suggest that it is applicable to recruit student facilitators when implementing virtual reality as a learning methodology, as they may contribute to participation and competence. Although the time spent on training student assistants may be a challenge [48], the use of peer supervision can address the time-related challenges in the implementation of virtual reality simulation in health education settings. However, when students have too many demands placed on their time, they are more likely to experience a high cost of engaging in the activity [49]. This may negatively influence their motivation to participate; therefore, it may be useful to focus on creating and facilitating realistic timeframes for the involved students (and faculty) when implementing virtual reality in health professions education.

Collaborative activities with students as stakeholders and student assistants may also help strengthen students’ competence in supervision, particularly if this is linked to formally obtaining supervisor competence [50]. Students normally do not have the same “expert knowledge” as faculty members, but it is conceivable that they may make a greater impact as motivators by virtue of being fellow students and relevant persons with whom other students can compare themselves. So far, little extant research has examined this, so it may be useful to explore this in future research. Common pedagogical solutions involving stakeholders may encourage employees, both internally and across universities and other academic institutions, to exchange experiences and inspire each other in a mutual learning process. This also has the potential to make pedagogical work easier [39].

#### Potential

A total of 7 categories emerged from the synthesis of the articles selected for this review, but we were unable to identify any links between them and the *potential* category, as May outlined [13]. *Potential* concerns individual interest, intention, and motivation and the collective valuation of and commitment to implementation. These processes are described as necessary antecedents for individual and collective behaviors [51-53] and, therefore, are crucial to the success of any implementation [20]. Without persistent individual and collective drive among the users of the innovation, it is unlikely that it will be sustained over time. Nevertheless, our findings demonstrate that aspects relating to *potential*—individual and collective agency and motivation—have not been emphasized in the existing literature on virtual reality implementation in health professions education [30-35]. The social-structural prerequisites (capacity and contribution) for implementation and aspects of the technology itself (capability) have received considerably more attention. However, given the importance of agency and motivation in successful implementation, we encourage the researchers involved in future studies of virtual reality implementation in health professions education contexts to include this crucial aspect of the process. Such studies may include mixed qualitative and quantitative data collection strategies, with their quality relying on their ability to combine different types of data in meaningful ways [54]. However, agency should be studied in the context of the specific implementation process in question. For example, in line with Ajzen [51] and expectancy-value theory in general [55], faculty members are unlikely to be motivated to implement new technologies or teaching methods unless they perceive that the innovation has practical value. Thus, key stakeholders, such as faculty members, should be invited to participate from the start of the development process to ensure that the innovation’s educational content is valued.

### Limitations

This review has several limitations. First, it is possible that relevant literature was not included in this review, although several databases and government web pages were searched. We could have broadened our understanding of virtual reality and used other keywords (eg, “augmented reality” and “computer simulation”) to obtain a wider overview of the existing studies. However, because of the scope of this study and the definition presented in the *Introduction*, we chose only immersive virtual reality in health professions education. This could be viewed as a limitation, as we excluded several articles that described virtual reality in a manner different from our definition and in other educational contexts. The concept of virtual reality is used in many ways, which poses a challenge for drawing conclusions based on virtual reality research. Having our definition of virtual reality broadened could have led us to include more articles, which might have influenced our findings. Moreover, it is a challenge when searching databases that the term virtual reality includes very different technologies. A common definition and use of *virtual reality* would be of value for the evidence base.

Furthermore, the quality of the included studies was not assessed as part of this scoping review because a scoping study does not seek to assess evidence quality and, consequently, cannot determine whether studies provide robust or generalizable findings [27,28]. However, this should be mentioned as a limitation of this study.

Moreover, searching for only English- and Norwegian-language papers limited this review’s findings. However, this choice was made after careful consideration. Because of the language knowledge in the research group, we conducted initial hand searches on Eastern European government web pages (Serbian, Croatian, and Bosnian Ministry of Education Government web pages) and in the Directory of Open Access Journals, using the keywords *“*Implementacija I/ili virtulane stvartnosi I/ili zdrastvenom strucnom obrazovanju*.*” Hand searches using these keywords were also conducted in 2 Eastern European scientific journals (*Hrcak* and *Nacionalna i sveucilisna knjiznica u Zagrebu*) on February 18, 2022. Owing to a lack of findings, these searches were excluded from the *Methods* section. We decided that focusing on the Norwegian and English languages was more relevant, as the project from which this scoping review was derived was conducted in a Norwegian higher education context [21].

### Conclusions

This scoping review has provided an overview of the sparse literature on virtual reality implementation in health professions education. The included articles provided recommendations concerning collaborative participation, availability, expenses, guidelines, technology, careful design and evaluation, and training. These aspects can be connected to the 4 constructs in May’s theory of implementation and are important to consider when planning virtual reality implementation in health professions education.

Recommendations for virtual reality implementation in health professions education aim to ensure faculty’s and students’ competence with the latest technology. By securing well-planned training for both faculty and students, cognitive abilities can be improved. Collaborative participation by including end users in the development process can ensure the successful implementation of virtual reality in higher education contexts. To secure motivation and stakeholders’ potential for using virtual reality, faculty and students could be invited to participate from the start of the development process to ensure that the innovation’s educational content is valued. Moreover, technological challenges and usability issues should be resolved before implementation to ensure that pedagogical content is the focus, and not the technical barriers. This accentuates the importance of piloting, sufficient time resources, basic testing, and sharing of experiences before implementation. Furthermore, implementing virtual reality in education is currently expensive and time-consuming; therefore, cost-benefit analyses may be of value.

On the basis on our findings, virtual reality implementation in health professions education is a new and underexplored research field. As we could not identify results concerning potential, we argue that more studies investigating individual interest, intention, and motivation, as well as the collective valuation of and commitment to virtual reality implementation, are needed, as individual engagement is also crucial in implementation processes. Moreover, because of the scant research in this area, future research could further investigate viable and effective strategies for implementing virtual reality in health professions education. Finding a common definition and use of the term *virtual reality* would also be of value for the evidence base, as this would make it easier to examine implementation processes using similar education measures.

## Appendix

Multimedia Appendix 1 PRISMA-ScR (Preferred Reporting Items for Systematic Reviews and Meta-Analyses Extension for Scoping Reviews) checklist.

Multimedia Appendix 2 Search strategy followed in CINAHL, Education Source, Academic Search Elite, and PubMed.

## Abbreviations

PRISMA: Preferred Reporting Items for Systematic Reviews and Meta-Analyses
