# Supplementary material for: Implementation of Virtual Reality in Health Professions Education: Scoping Review
Source: JMIR Med Educ. 2023 Jan 24;9:e41589. doi: 10.2196/41589 (PMC9906320; doi:10.2196/41589)
Supplement: Multimedia Appendix 2 [file mededu_v9i1e41589_app2.docx]

Multimedia Appendix II

**Search strategy in CINAHL:**

| **#** | **Query** | **Results** |
| --- | --- | --- |
| S9 | S1 AND S4 AND S7 (Limiters - Published Date: 20170101-20221231 Expanders - Narrow by Language: - english). | 299 |
| S8 | S1 AND S4 AND S7 | 792 |
| S7 | S5 OR S6 | 290,256 |
| S6 | SU “Implementation science” OR SU “learning methods” OR SU “program implementation” OR SU “teaching methods” | 78,496 |
| S5 | implement* or “implementation science” or “implementation method” or “implementation strateg*” or “program implementation” or “training programs” | 246,554 |
| S4 | S2 OR S3 | 307,508 |
| S3 | MH “Education, health sciences+” | 290,058 |
| S2 | “higher education (health)” or “nursing school” or “health scienc* educat*” or “allied health education” or “medical educat*” or “social science* educat*” or “healthcare education” or “health occupation students” | 63,920 |
| S1 | MH «computer simulation+» | 25,129 |

**Search strategy in Education source:**

| **#** | **Query** | **Results** |
| --- | --- | --- |
| S11 | S4 AND S7 AND S10 Limiters - Published Date: 20170101-20211231. Expanders - Apply equivalent subjects. Narrow by Language: - english. Search modes - Boolean/Phrase | 108 |
| S10 | S8 OR S9 | 214,045 |
| S9 | KW ( (“program implementation” or “program design” or “program development” or “educational programs”) ) OR SU ( (“program implementation” or “program design” or “program development” or “educational programs”) ) | 26,581 |
| S8 | (Implementation or implement* or “implementation science” or “implementation method” or “implementation strateg*” or “program implementation” or “training programs”) | 213,169 |
| S7 | S5 OR S6 | 209,090 |
| S6 | (“higher education (health)” or “nursing school” or “health scienc* educat*” or “allied health education” or “medical educat*”, “social science* educat*”) | 20,338 |
| S5 | KW (“medical education” or “healthcare education” or “higher education”) OR SU (“medical education” or “healthcare education” or “higher education”) | 191,747 |
| S4 | S1 OR S2 OR S3 | 23,624 |
| S3 | KW computer simulation OR TX "computer simulation" OR TX "virtual reality" | 23,013 |
| S2 | AB "virtual reality" OR KW "virtual reality" | 3,203 |
| S1 | AB vr OR TI vr | 1,710 |

**Search strategy in Academic search elite:**

| **#** | **Query** | **Results** |
| --- | --- | --- |
| S10 | S3 AND S6 AND S9 (Limiters - Published Date: 20170101-20221231. Narrow by Language: - english) | 48 |
| S9 | S7 OR S8 | 950,876 |
| S8 | Implementation OR “program implementation” | 437,365 |
| S7 | implement* or “implementation science” or “implementation method” or “implementation strateg*” or “program implementation” or “training programs” | 950,876 |
| S6 | S4 OR S5 | 502,010 |
| S5 | “Medical education” or “higher education” | 474,913 |
| S4 | “higher education (health)” or “nursing school” or “health scienc* educat*” or “allied health education” or “medical educat*”, “social science* educat*” or “healthcare education” or “health occupation students” | 31,052 |
| S3 | S1 OR S2 | 21,497 |
| S2 | TI "virtual reality" OR AB "“virtual reality” OR TI "VR" OR AB "VR" | 21,436 |
| S1 | “Virtual reality in higher education” or “simulation methods and models” | 79 |

**Search strategy in PubMed:**

| # | Query | Results |
| --- | --- | --- |
| S1 | (("virtual reality"[All Fields] OR "VR"[All Fields]) AND ("higher education"[All Fields] OR "higher education health"[All Fields] OR "medical education"[All Fields] OR "health science education"[All Fields] OR "nursing school"[All Fields] OR "healthcare education"[All Fields] OR "allied health education"[All Fields] OR "social science education"[All Fields] OR "health occupation students"[All Fields]) AND ("implementation"[All Fields] OR "implementation science"[All Fields] OR "implementation method"[All Fields] OR "implementation strategies"[All Fields] OR "program implementation"[All Fields] OR "training programs"[All Fields]) Filters: from 2017 - 2022 | 94 |
